# Supplementary figures and images for: Development and characterization of fourteen novel microsatellite markers for the chestnut short-tailed fruit bat (Carollia castanea), and cross-amplification to related species
Source: PeerJ. 2016 Sep 20;4:e2465. doi: 10.7717/peerj.2465 (PMC5036068; doi:10.7717/peerj.2465)

CC-7

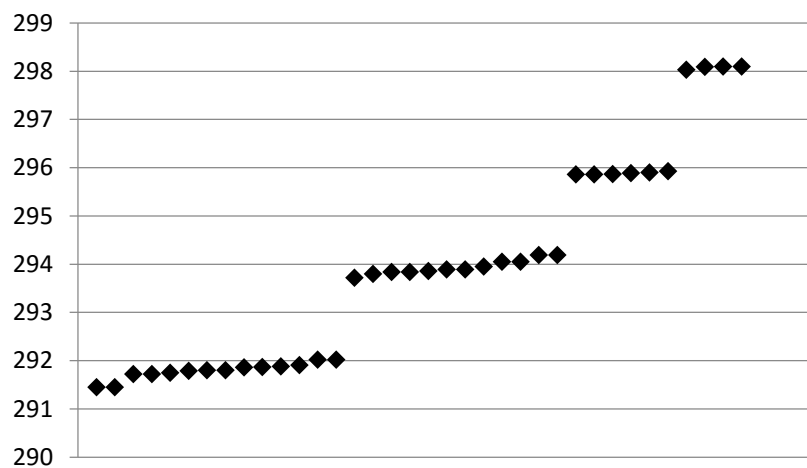

CC-10

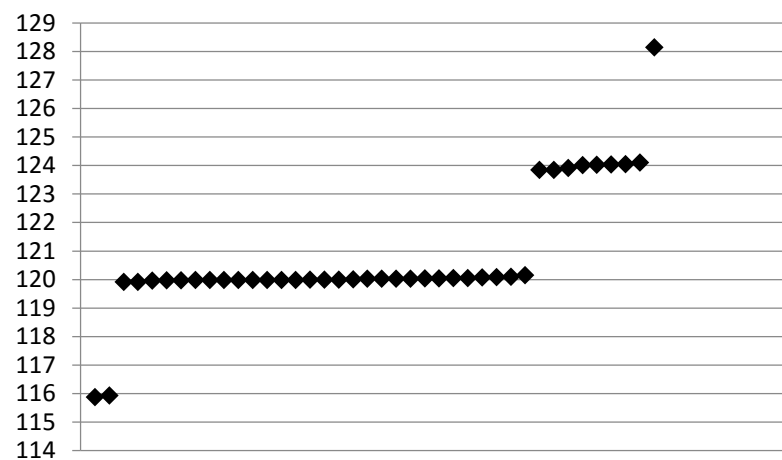

CC-13

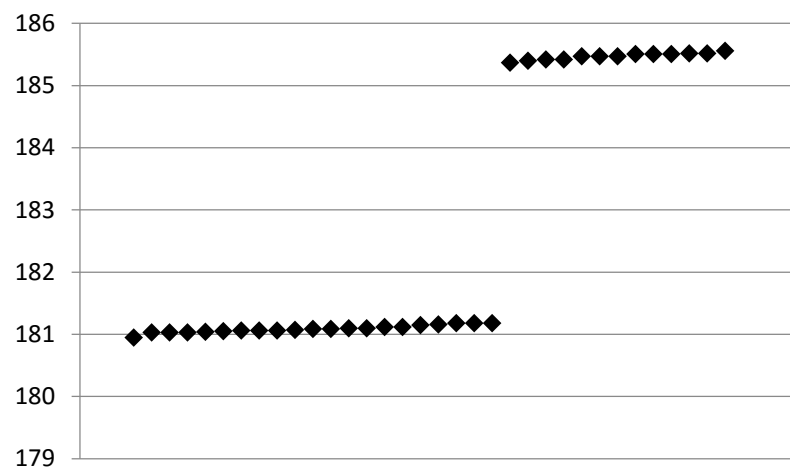

CC-18

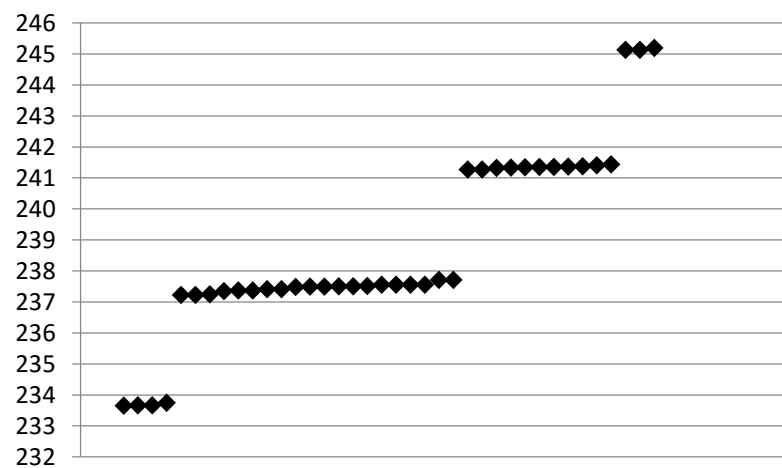

CC-19

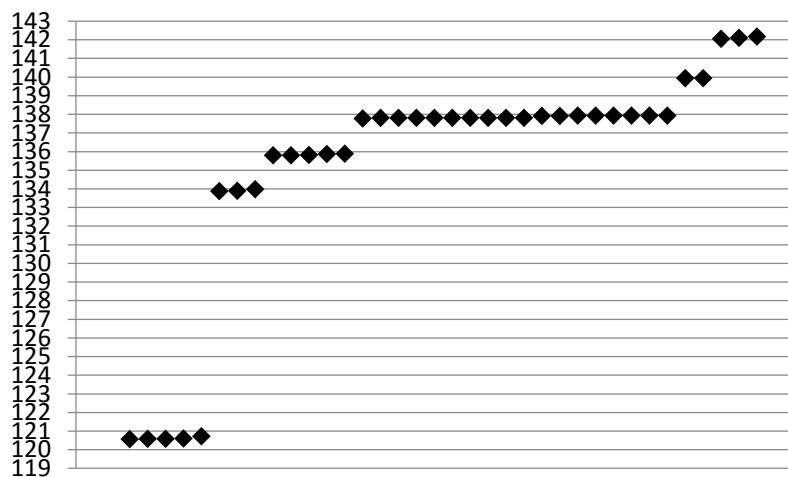

CC-23

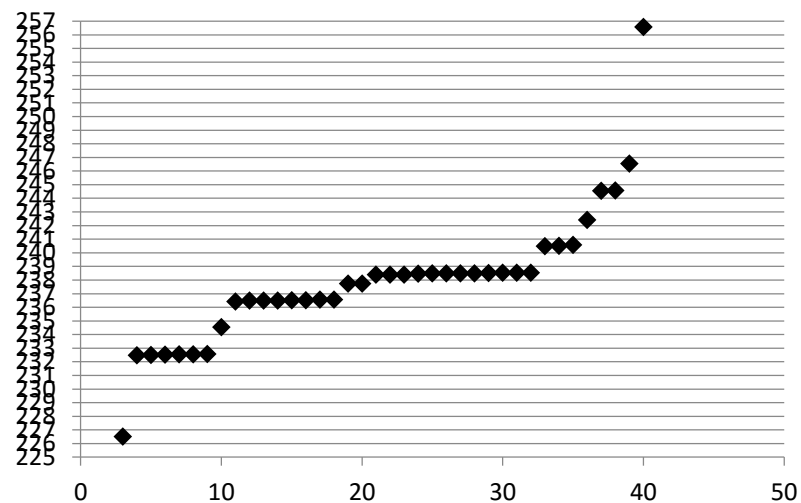

CC-25

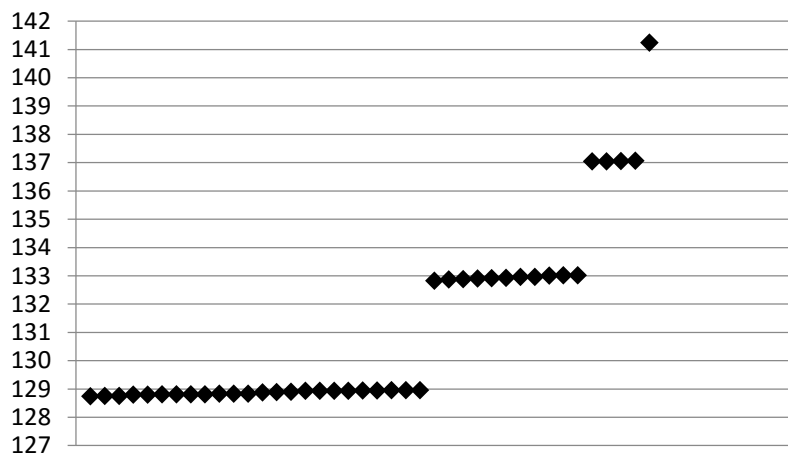

CC-26

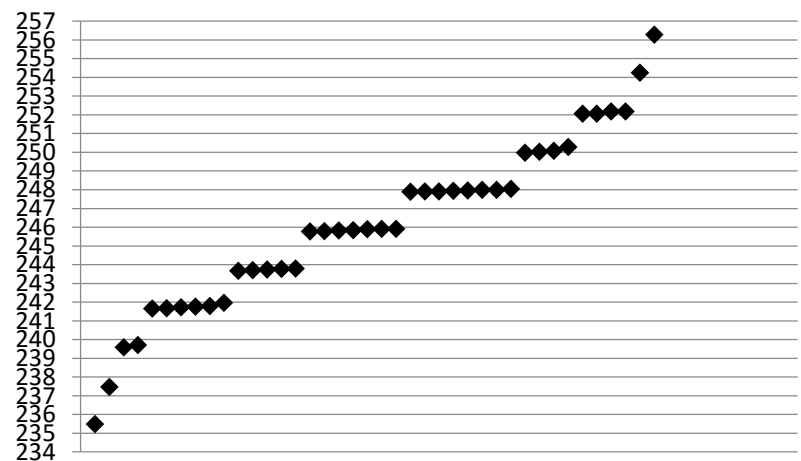

CC-27

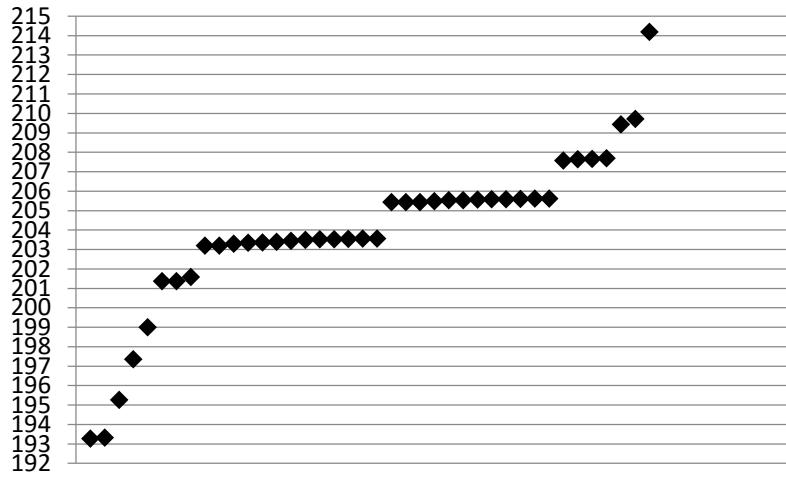

CC-29

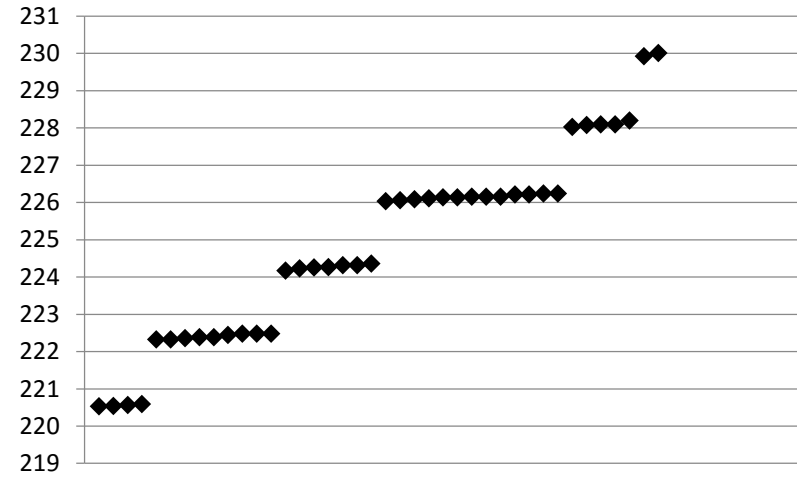

CC-30

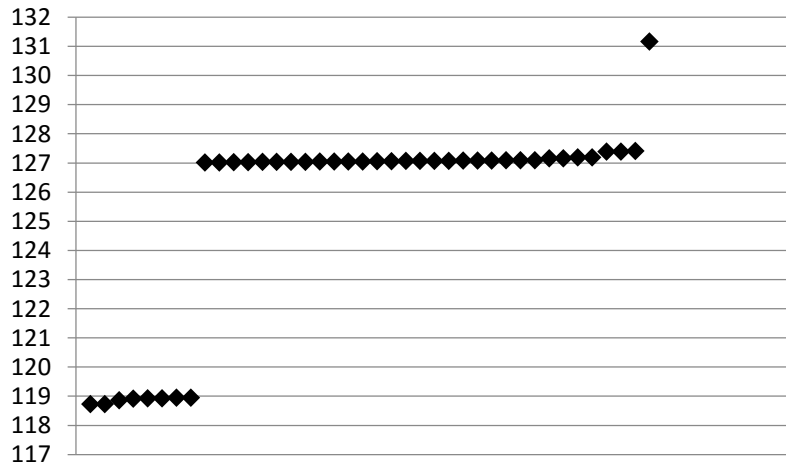

Supplement: Figure S1 — Distribution of raw allele size for each of the fourteen microsatellite loci, across all 20 individuals of Carollia castanea. For all charts allele size is listed on the y-axis. [file peerj-04-2465-s001.pdf]
